# Symptomatic Massive Splenomegaly in Persistent Polyclonal B-cell Lymphocytosis Requiring Splenectomy

**Published:** 2015-06-29

**Authors:** Shanel B. Bhagwandin, Elliot S. Weisenberg, Howard Ozer, Ajay V. Maker

**Affiliations:** 1 Department of Surgery, University of Illinois at Chicago Medical Center, Chicago, Illinois; 2 Department of Pathology, University of Illinois at Chicago Medical Center, Chicago, Illinois; 3 Section of Hematology and Oncology, Department of Medicine, University of Illinois at Chicago Medical Center, Chicago, Illinois

## Abstract

**Introduction:**

Persistent polyclonal B-cell lymphocytosis (PPBL) is a rare lymphoproliferative hematological disease characterized by binucleated lymphocytes, CD 19^+^ CD 5^−^lymphocytosis, and elevated levels of serum immunoglobulin M (IgM). It can rarely be associated with splenomegaly, though the disease usually remains indolent.

**Case Presentation:**

We present a case of PPBL in a young man with massive splenomegaly that mimicked isolated splenic lymphoma requiring splenectomy for persistent pain, symptoms, and diagnosis.

**Discussion:**

Determining the etiology of splenomegaly in these patients is often confounding due to a lack of a tissue diagnosis and the limited morphological and immuno-histochemical features of PPBL, therefore, the presentation remains highly concerning for lymphoma.

**Conclusion:**

The presentation, surgical treatment, tissue and peripheral blood molecular analysis, and flow cytometry integral to managing these patients and to prevent an assumptive and misleading diagnosis are reviewed.

## Background

**Figure 1:**
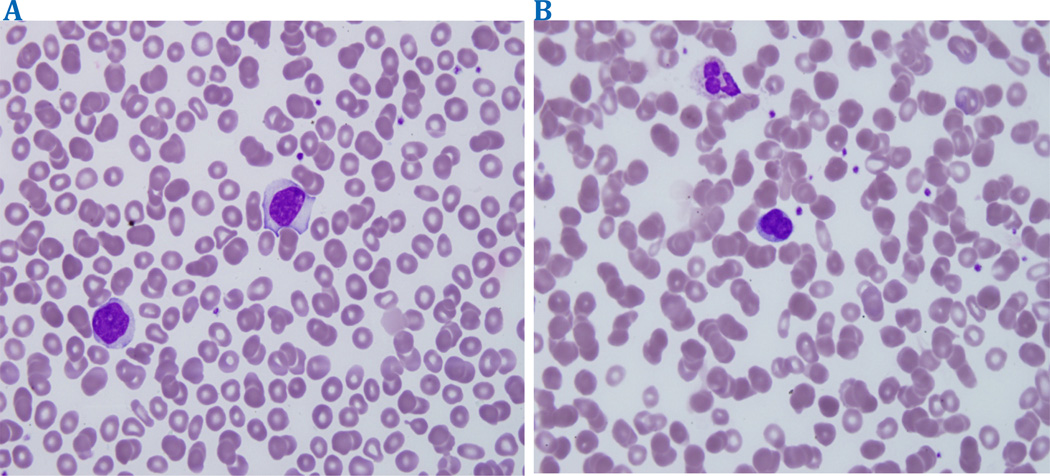
Blood smear comparing normal mononuclear lymphocytes (a) and binucleated lymphocytes (b) found in our patient with splenomegaly.

Persistent polyclonal B-cell lymphocytosis (PPBL) is an extremely rare, lymphoproliferative hematological disease characterized by atypical binucleated lymphocytes on peripheral blood smear. Lymphocytosis is typically CD 19^+^ and CD 5^−^ with predominance of polyclonal serum immunoglobulin M (IgM) by immunohistochemistry and gene rearrangement, respectively. The total lymphocyte count is not always elevated, but the presence of polyclonal binucleated lymphocytes typically distinguishes this entity from that of other B-cell chronic lymphoproliferative disorders (Figure 1)[1,2].

PPBL has been most frequently described among middle-aged women who smoke, and may be associated with asymptomatic mild splenomegaly and lymphadenopathy in approximately 10% of cases. This clinical finding is the most common, albeit atypical, as the course of the disease usually remains indolent. Some patients have experienced intermittent or chronic fatigue as a notable post-viral syndrome. Massive splenomegaly is exceedingly rare[2].

We present a case of a young male smoker who progressed to develop massive splenomegaly warranting splenectomy for both diagnosis and symptomatic relief. The importance of correlating the clinical presentation with molecular analysis, flow cytometry, and the identification of binucleated lymphocytes on peripheral blood smear are integral to prevent an assumptive and misleading diagnosis[2].

## Case presentation

A 25 year-old male presented to his urologist with an acute onset of urinary frequency and nocturnal incontinence of 2 months duration. Additional symptoms included chronic fatigue and suprapubic and lower abdominal pain. He did not have any associated dysuria or hematuria. He denied notable bruising, bleeding, fevers, or chills, but did experience occasional night sweats.

The patient smoked marijuana twice daily and was a former tobacco smoker. He had an unremarkable past medical history, and a family history significant for two uncles diagnosed with lymphoma.

**Figure 2:**
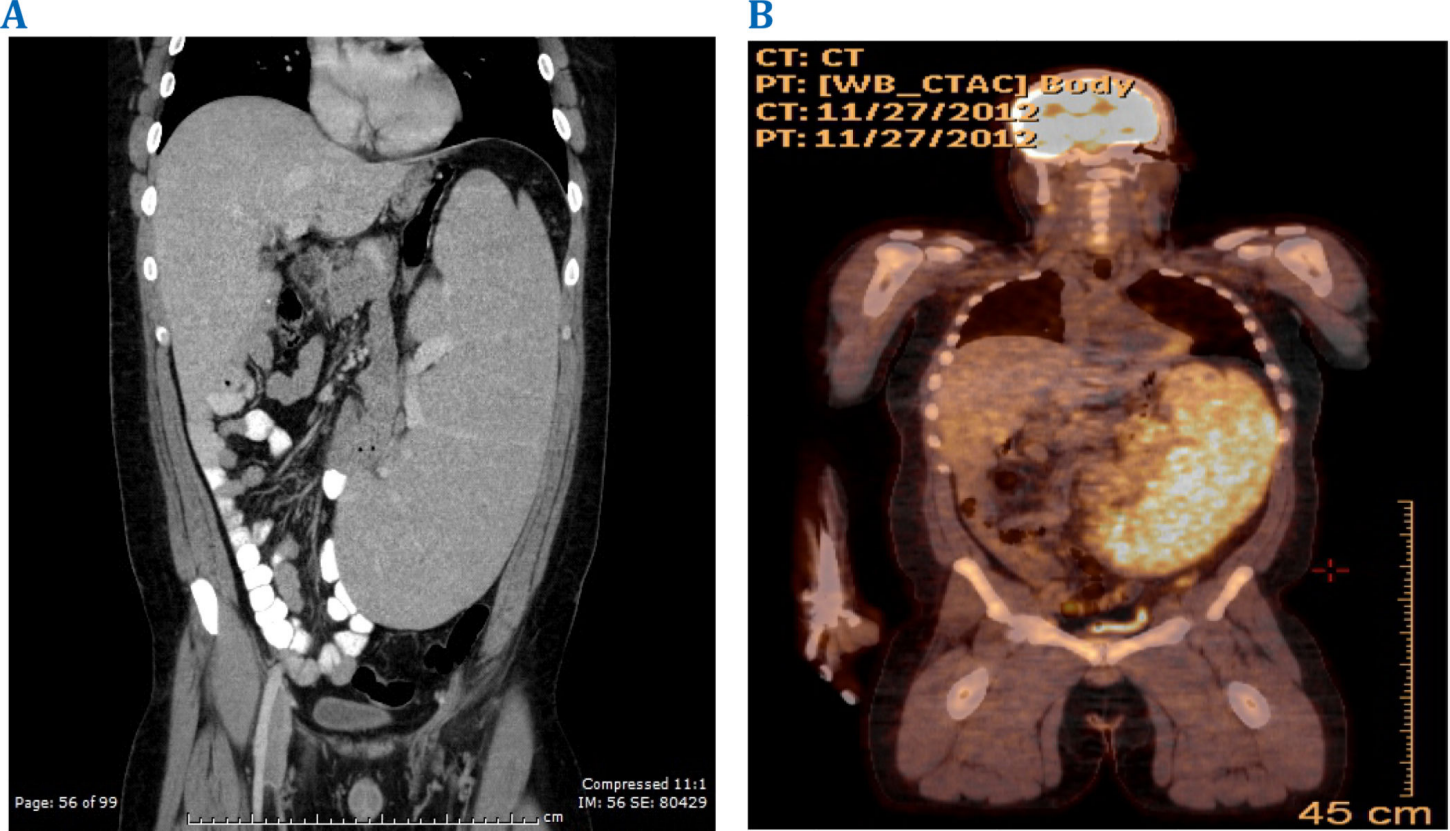
CT and PET imaging reveal massive splenomegaly.

Physical exam was significant for a large mass extending from the anterior midline to the left costovertebral angle in anterior-posterior dimensions, and from the left subcostal margin to the pelvis in length. Manipulation incited suprapubic discomfort. There was no associated lymphadenopathy. His initial complete blood cell count (CBC) showed isolated lymphocytosis of 6.1 × 10^9^/L and thrombocytopenia with platelets of 115 × 10^9^/L. The peripheral blood smear demonstrated rare atypical binucleated lymphocytes. His initial work-up included an abdominal ultrasound (US) and subsequent computed tomography (CT) scan of the chest, abdomen, and pelvis that demonstrated massive splenomegaly approximating 35cm. The lower pole of the spleen was significantly compressing the bladder. A PET scan revealed isolated uptake in the enlarged spleen with an SUV of 6.6. (Figure 2)

These findings prompted a bone marrow biopsy for lymphoma staging that demonstrated binucleated lymphocytes indicative of a B-cell lymphoproliferative disorder/lymphoma. Flow cytometry, surface markers, and molecular studies of bone marrow lymphocytes did not demonstrate evidence of clonality by either method.

Given his progressive symptoms, thrombocytopenia, abdominal discomfort, massive splenomegaly, and concern for splenic lymphoma without definitive pathology, the patient underwent a splenectomy for diagnostic and therapeutic purposes following scheduled meningococcal, pneumococcal, and H. influenza vaccinations. He had an uncomplicated post-operative course with resolution of his fatigue, pain, and urinary continence.

## Results

### Operative Details

**Figure 3:**
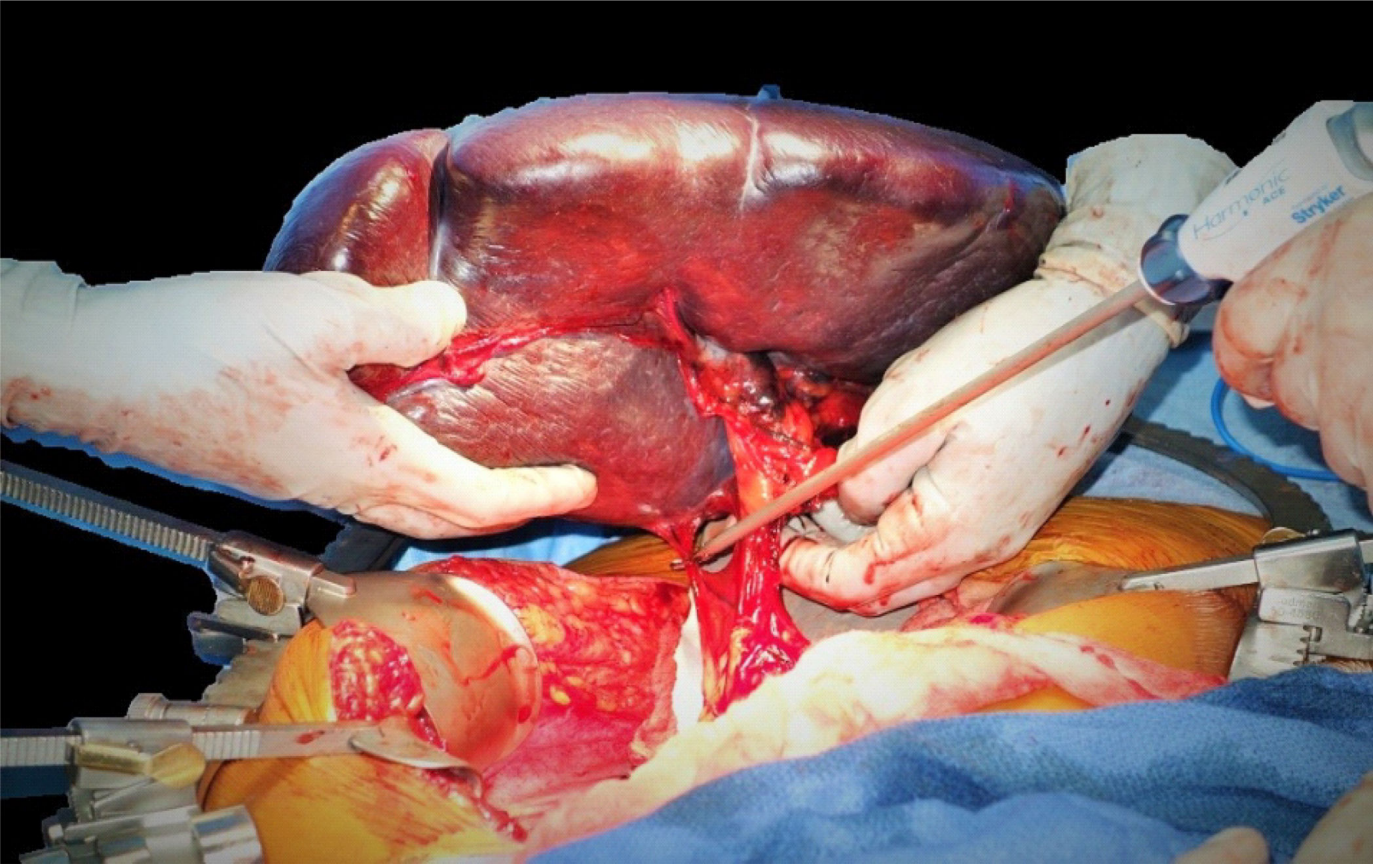
Intraoperative image demonstrating delivery of the enlarged spleen from the patient's abdomen after division of the splenic hilum and vascular ligation.

**Figure 4:**
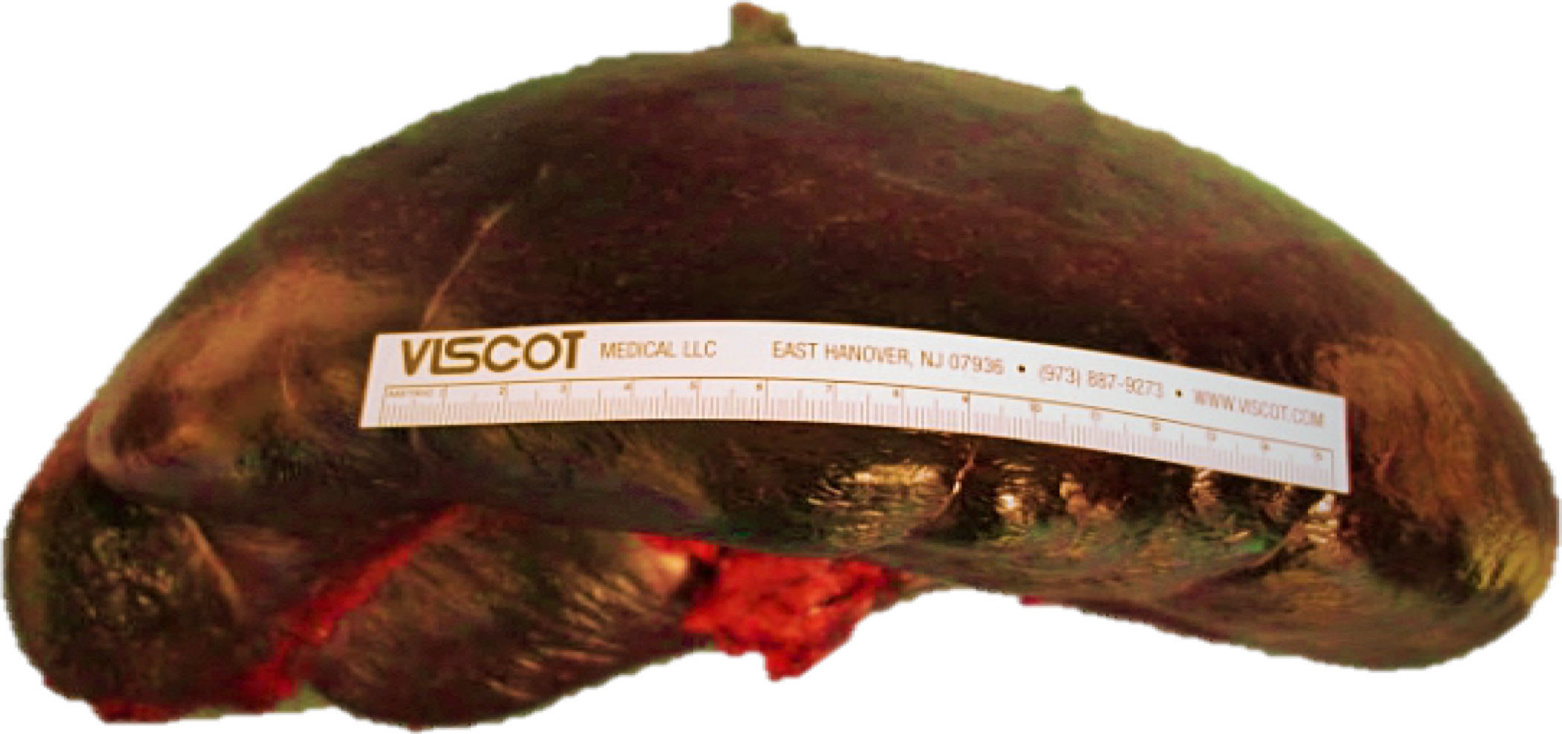
Gross pathologic specimen after autotransfusion of blood measuring 36.4 × 15.3 × 10.6 cm and weighing 4082 gm.

Though most splenectomies are performed laparoscopically in our surgical unit, we determined that a spleen of this size and weight would be better approached through an open resection. The splenic artery was isolated and ligated to allow auto transfusion and drainage of blood volume through the splenic vein to shrink the heavy and difficult to manipulate spleen. After drainage of significant blood volume and resulting size decrease, the splenic vein was divided along. The splenic ligaments were stretched and distorted due to the increased size of the organ, and were sequentially divided. Blood loss was minimal. The excised specimen weighed 4082 gm and measured 36.4×15.3×10.6 cm after auto transfusion (Figures 3, 4). The patient was discharged on post-operative day 3 without any peri or post-operative complications.

### Histopathology analysis

There was prominent white pulp expansion of B lymphocytes in the germinal centers, mantle zones, and marginal zones extending into the red pulp. These cells were not clonal by flow cytometry or immunoglobulin gene rearrangement studies. A panel of immunostains was negative for CD5, CD19, BCL 2, and HHV 8; as was an in situ hybridization study for Epstein Barr virus. Regional lymph nodes revealed follicular hyperplasia, a sinus histiocytosis pattern, and reactive germinal centers. Conventional cytogenetics revealed normal karyotype and negative gene rearrangements. The i(3q) chromosomal abnormality was negative and HLA-DR7 was not detected.

### Discussion

Persistent and absolute lymphocytosis is attributed to chronic lymphoproliferative disorders of B cells that include chronic lymphocytic leukemia (CLL), prolymphocytic leukemia (PLL), splenic lymphoma, hairy cell leukemia (HCL), and mantle cell lymphoma (MCL). These dyscrasias are predominantly the result of progressive expansion of an abnormal clonal population of B lymphocytes in the blood, bone marrow, or tissues. All cells within the clonal population display the same unique Ig gene rearrangement and demonstrate a consistent chromosomal abnormality[3].

In 1982, a persistent polyclonal lymphocytosis of binucleated B lymphocytes was first reported in three women by Gordon et al. The disorder is most commonly diagnosed by the presence of binucleated lymphocytes as well as an increased polyclonal serum IgM level. It was theorized that the process was reactive to the abnormal stimulation of B-cells that was inherent among individuals with an underlying genetic predisposition. This study identified the human leukocyte antigen HLA-DR7 in all 3 patients. Through molecular and immunological analysis, this HLA haplotype has been identified among 26% of the Caucasian population. Troussard et al. later described an additional isochromosome for the long arm of chromosome 3, +i(3q), in 6 cases and premature chromosome condensation (PCC) in all 7 cases of their series. Additionally, reports of familial PPBL and a case of PPBL occurring among monozygotic twins have also suggested this strong genetic predisposition[3,7].

In a series of 25 patients with PPBL, 77% were found to have a +i(3q) recurrent chromosomal abnormality, whereas in the largest reported series of 111 patients, only 34% of patients harbored this mutation. Furthermore, i(3q) is observed exclusively in non-binucleated cells, which may account for its low accuracy as a tumor marker considering that the population of lymphocytes may be primarily replaced by binucleated cells at the time of diagnosis[2,10].

Although the etiology of PPBL has not been elucidated, an association with tobacco smoking in the development of the polyclonal lymphocytosis has been considered. In the largest series of 111 patients, 98% were smokers and two of the patients had a decrease of lymphocytosis and the number of binucleated lymphocytes after smoking cessation. Isochromosome +i(3q), however, did persist throughout the 2 years follow-up after tobacco use[2,10].

Splenomegaly is rare in patients with PPBL though there are reports of progressive spleen enlargement up to six years following diagnosis of PPBL. A series of 5 patients with splenomegaly with PPBL presented by Del Giudice et al. found that 3 of those patients had similar immunophenotypes of peripheral lymphocytes including expression of BCL-2 and IgH rearrangements. They noted HLA-DR7 in all five cases. These patients had an otherwise indolent clinical presentation and course of disease progression[2].

The long-term follow-up of 111 patients with typical PPBL revealed that 89% of patients were symptom free after a median follow-up of 4.4 years. Two patients developed lung cancer, one of whom died 9 years after diagnosis. Three additional cases of non-Hodgkins Lymphoma (NHL) were observed as well as one case of cervical cancer, however, none of the patients in the series developed significant splenomegaly warranting splenectomy[10].

Considering the long-term follow-up and event-free survival in these patients, any aggressive medical or surgical intervention should be limited once a diagnosis has been made. Awareness of PPBL, a high index of suspicion, and skepticism of a malignant diagnosis of a B-cell lymphoproliferative disorder in the absence of immunophenotypic or molecular evidence of clonality, splenectomy may be necessary to render the correct diagnosis. The appropriate management of patients with PPBL relies on differentiating these patients from those with other chronic lymphoproliferative B-cell disorders, secondary malignancies, or lymphoma; and as a result close follow-up is recommended.

## Conclusions

The PPBL patient reported here had an indolent course, but presented due to worsening symptoms of massive splenomegaly causing a mass effect on surrounding organs. This case supports past reports of PPBL associated with smoking and splenomegaly, though growth to this size and weight appears exceedingly rare. Splenic resection was critical to establish the correct diagnosis, and this case highlights that PPBL can present to the surgeon only with binucleated lymphocytes and massive splenomegaly without other hematologic genetic abnormities or being HLA-DR7+.

## Keypoints

Persistent polyclonal B-cell lymphocytosis (PPBL) is a rare lymphoproliferative hematological disease characterized by binucleated lymphocytes.

Massive splenomegaly mimicked isolated splenic lymphoma in this patient requiring splenectomy for persistent pain, symptoms, and diagnosis.

## References

[1] Tonelli S, Petronilla V, Sacchi S (2010). Persisistent polyclonal B lymphocytosis: morphological, immunological, cytogenetic and molecular analysis of an Italian case. Leuk Res.

[2] Del Giudice I, Pileri SA, Rossi M (2009). Histopathological and molecular features of persistent polyclonal B-cell lymphocytosis (PPBL) with progressive splenomegaly. Br J Haematol.

[3] Sun P, Juskevicius R (2012). Histological and immunohistochemical features of the spleen in persistent polyclonal B-cell lymphocytosis closely mimic splenic B-cell lymphoma. Diagnostic Pathology.

[4] Agrawal S, Matutes E, Voke J (1994). Persistent polyclonal B-cell lymphocytosis. Leuk Res.

[5] Gordon DS, Jones BM, Browing SW (1982). Persistent polyclonal lymphocytosis of B-lymphocytes. NE Journal of Medicine.

[6] Troussard X, Cornet E, Lesesve JF (2007). Polyclonal B-cell lymphocytosis with binucleated lymphocytes (PPBL). OncoTargets and therapy.

[7] Mossafa H, Malaure H, Maynadie M (1999). Persistent polyclonal B lymphocytosis with binucleated lymphocytes: a study of 25 cases. British Journal of Haematology.

[8] Himmelmann A, Ruegg R, Fehr J (2001). Familial persistent polyclonal B-cell lymphocytosis. Leuk Lymphoma.

[9] Carr R, Fishlock K, Matutes E (1997). Persistent polyclonal B-cell lymphocytosis in identical twins. Br J Haematol.

[10] Cornet E, Lesesve JF, Mossafa H (2009). Long-term follow-up of 111 patients with persistent polyclonal B-cell lymphocytosis with binucleated lymphocytes. Leukemia.

[11] Carstairs KC, Francombe WH, Scott JG (1985). Polyclonal lymphocytosis of B-lymphocytes induced by cigarette smoking?. Lancet.

